# UAV-YOLO: Small Object Detection on Unmanned Aerial Vehicle Perspective

**DOI:** 10.3390/s20082238

**Published:** 2020-04-15

**Authors:** Mingjie Liu, Xianhao Wang, Anjian Zhou, Xiuyuan Fu, Yiwei Ma, Changhao Piao

**Affiliations:** 1 Department of Automation, Chongqing University of Posts and Telecommunications, No. 2 Chongwen Road, Chongqing 40000, China; liumj@cqupt.edu.cn (M.L.); wangxhcqupt@163.com (X.W.); mayw@cqupt.edu.cn (Y.M.); 2 Chongqing Changan New Energy Science and Technology Co., Ltd., Chongqing 401120, China; zhouanjian@changan.com.cn; 3 Chongqing SPIC ZINENG Technology Co., Ltd., Chongqing 404100, China; fuxiuyuan@spic.com.cn

**Keywords:** unmanned aerial vehicle, object detection, convolutional neural network

## Abstract

Object detection, as a fundamental task in computer vision, has been developed enormously, but is still challenging work, especially for Unmanned Aerial Vehicle (UAV) perspective due to small scale of the target. In this study, the authors develop a special detection method for small objects in UAV perspective. Based on YOLOv3, the Resblock in darknet is first optimized by concatenating two ResNet units that have the same width and height. Then, the entire darknet structure is improved by increasing convolution operation at an early layer to enrich spatial information. Both these two optimizations can enlarge the receptive filed. Furthermore, UAV-viewed dataset is collected to UAV perspective or small object detection. An optimized training method is also proposed based on collected UAV-viewed dataset. The experimental results on public dataset and our collected UAV-viewed dataset show distinct performance improvement on small object detection with keeping the same level performance on normal dataset, which means our proposed method adapts to different kinds of conditions.

## 1. Introduction

Object detection in Unmanned Aerial Vehicle (UAV), as a kind of burgeoning technique, has numerous applications, such as aerial image analysis, intelligent surveillance, and routing inspection [1,2,3,4]. Object detection has recently experienced a lot of progress. Especially with the development of large-scale visual datasets and increased computation power, the deep neural network (DNN)—particularly, the convolutional neural network (CNN) [5]—has demonstrated record breaking performance in computer vision tasks including object detection [6,7,8]. However, it is still a challenging work due to special perspective.

Object detection can be divided into traditional handcrafted feature-based object detection [9,10] and deep-learning-based object detection [11,12]. It focuses on the target-feature extraction method design for handcrafted feature-based object detection; however, it is still hard to satisfy different conditions, which leads to most of these kinds of methods just being used for limited environment [13,14,15]. On the other hand, with the development of computation hardware, deep-learning-based methods can not only enhance the accuracy but also realize real-time detection.

Although deep-learning-based approaches have contributed to a great deal of progress in object detection, the issues of miss-detection still occur in UAV. The causes of these issues can be mainly attributed to the following: (i) the receptive field of network is not robust enough to small objects; (ii) the training dataset is limited to UAV perspective. In general, object feature representation and corresponding training dataset are essential for improving the performance of object detection. Besides, accuracy and processing time trade-off is also important to real-world applications.

Encouraged by these problems, we develop an object detection method based on You only look once (YOLO) [16], which focus on small object detection, named as UAV-YOLO. To improve detected performance on small objects, we collect the dataset based on UAV view and improve network of YOLO to enlarge the receptive field. In particular, the contributions of our study are as follows: (i) create a UAV perspective-based human detection dataset which can be used to improve the performance on human detection; (ii) improve network structure of YOLO to enlarge the receptive field, further, to enhance the performance on small human detection.

The remainder of this paper is organized as follows: Section 2 introduces related work and Section 3 describes the proposed method in detail. Section 4 demonstrates the experimental result and contains a discussion on specific comparison analysis. Section 5 provides concluding remarks.

## 2. Related Work

A deep-learning-based detector can be divided into two categories: two-stage and one-stage. RCNN family (RCNN [17], Fast RCNN [18], and Faster RCNN [19]) is a two-stage algorithm that outperforms numerous other detection algorithms in terms of accuracy. However, these kinds of approaches need more computational cost which results in the consumption on processing time. From the one-stage detector perspective, single-shot multibox detector (SSD) [20] and You only look once (YOLO) are proposed by considering both accuracy and processing time. Especially YOLO can balance the performance on accuracy and processing time well.

RCNN adopts a region-proposal-based strategy [21] in which each proposal is scale-normalized before classifying with a ConvNet [22]. More accurate detectors such as Fast RCNN and Faster RCNN advocate using features computed from a single scale, since it offers a good performance on balancing accuracy and processing time. However, it still cannot satisfy requirement to embedded board on processing time. In addition, due to large amount of memory taken up and the complexity of the network, it is hard to be used on Rotorcraft UAV [23,24].

Considering the high-efficiency, one-stage object detection attracts more attention, Liu et al. proposed the SSD method, which spreads out anchors of different scales to multilayers with ConvNet and enforces each layer to predict object at a certain scale. Fu et al. [25] proposed a deconvolutional single-shot detector (DSSD), which combines Residual-101 [26] with SSD and augments them with deconvolution layers to introduce additional large-scale context for object detection, improving accuracy. Li et al. [27] proposed a feature fusion single-shot multibox detector (FSSD) to enhance SSD with a novel and lightweight feature fusion module. They concatenate features from multiple layers at different scales, followed by downsampling blocks to generate new feature pyramids, which are fed to multibox detectors to predict final detected results. YOLO uses a single feedforward convolutional network to predict object categories and locations, which can arrive at 45 fps. Then, YOLOv2 [28] is proposed to improve YOLO in several aspects, such as using high-resolution layers, adding batch normalization on each convolution layer, and employing convolution layers with anchor boxes to predict bounding boxes instead of fully connected layers. With the development of basic network, YOLOv3 [29]—whose accuracy for human detection can reach 76% on VOC dataset [30]—is proposed by replacing backbone network with darknet-53 and employing multiscale features to detect the object. However, it still cannot work well on UAV-viewed (small) object detection due to lack of corresponding training data and limited receptive field.

On UAV-viewed object detection aspect, deep-learning-based methods have already been widely applied. Ammour et al. [31] proposed a small-region-based detection method to detect vehicles. They use a deep CNN system as a feature extract tool, combined with a linear support vector machine (SVM) classifier to classify regions, which is obtained by segment input image into small homogeneous regions—into “car” and “no-car” classes. Bazi et al. [32] introduced a novel convolutional support vector machine (CSVM) network for UAV-viewed detection. The network is based on several alternating convolutional and reduction layers ended by a linear SVM classification layer. It relies on a set of linear SVMs as filter banks for feature map generation. To find a suitable feature space to solve the problem for small object detection in UAV view, Konoplich et al. [33] presented the adapted hybrid neural network, in which the last layers are divided into several blocks of variable size so that the network could extract features of different scales. Moreover, semantic segmentation based on contour-aware [34] and adverse method [35] is a good choice for small-sample learning, which is the key problem to UAV-viewed object detection. Jiang et al. [35] proposed a small-sample learning method via adversary. They built two submodels simultaneously based on the attribute of semantic classes for semantic segmentation discrimination. To precisely segment small objects, semantic classes are adversely modeled through computing the weighted costs based on the structural relationships between small samples and the others.

With the aim of developing a fast UAV perspective method for special uses of object detection, on one hand, we create a UAV-viewed dataset used for network training and test. On the other hand, we propose a UAV-viewed object detection method based on YOLOv3, called UAV-YOLO. To improve the performance of YOLOv3 on small object detection, collected data is first separated into “normal”, “far”, and “games” according to the distance and background in the environment. The model is then trained by collected data with data augmentation. During training, k-means [36,37] is used to cluster different numbers of anchor boxes to find the optimized number and size of them. Finally, the model is retrained by far-category data. On the other hand, Darknet-53—which is used as backbone network for YOLOv3—is also optimized to improve performance. We evaluate UAV-YOLO on both our collected UAV-viewed test dataset and classical VOC/COCO dataset; on the premise of keeping original YOLOv3 performance, our proposed UAV-YOLO can further enhance the performance on small object detection. In addition, it needs to be mentioned that we mainly focus on human detection.

## 3. Proposed Method

In our study, we collected a mass of UAV-viewed human dataset which will be introduced in Section 4.1.

We improve YOLOv3 by two different aspects: model training and backbone structure optimizing. YOLOv3 is first trained with data augmentation by the collected dataset that is divided into three categories (normal, far, and games) considering background clutters and distance between target and camera. K-means is conducted to optimize the number and size of anchor box during training. The backbone network is optimized to satisfy small object detection whilst keeping the performance unchanging on normal condition.

### 3.1. YOLOv3 Used for Human Detection on UAV Perspective

Given an image, it resizes to a fixed size (608×608) as an input to detector. Feature is extracted by darknet (backbone network of YOLOv3) whose final output is the 19×19×18 tensor of predictions. Assume that input image is separated into 7×7 grids. If the center of the target falls into a tile, that tile should be responsible for that detected target. Each tile predicts 2 bounding boxes and confidence scores for those boxes. These confidence scores reflect how confident the detector is that that box contains a target and also how accurate it thinks the box is that it predicts. Formally the confidence is defined as $Pr\left(object\right)\cdot IOU_{pred}^{truth}$. If no target exists in that tile, the confidence score should be zero; otherwise, it should equal the intersection over union (IOU) between predicted bounding box and ground truth. Each bounding box contains 5 predictions: center of bounding box relative to the bounds of the tile (x,y), the width and height related to the entire image (w,h), and confidence score. Finally, confidence prediction represents the IOU between predicted box and any ground truth box. Each tile also predicts *C* conditional class probabilities, $Pr\left(C_{i}|Object\right)$. These probabilities are conditioned on the tile containing a target. At test time, we multiply the conditional class probabilities and the individual box confidence predictions:

(1) $$Pr\left(C_{i}|target\right)\cdot \mathrm{Pr}\left(target\right)\cdot IOU_{pred}^{truth}=Pr\left(C_{i}\right)\cdot IOU_{pred}^{truth},$$

where $C_{i}$ is the *i*-th class the target belongs to. For evaluating the detector on our collected UAV dataset, we use i={1,2,3} since it has 3 labeled classes.

In YOLOv3 training, cross entropy is introduced as loss function. The format of loss function is as follows:

(2) $$\begin{array}{c} loss=\lambda_{coord}\sum_{i=0}^{S^{2}}\sum_{j=0}^{B}l_{ij}^{obj}\left[{\left(x_{i}-{\hat{x}}_{i}\right)}^{2}+{\left(y_{i}-{\hat{y}}_{i}\right)}^{2}\right]\\ +\lambda_{coord}\sum_{i=0}^{S^{2}}\sum_{j=0}^{B}l_{ij}^{obj}\left[{\left(\sqrt{\omega_{i}}-\sqrt{{\hat{\omega }}_{i}}\right)}^{2}+{\left(\sqrt{h_{i}}-\sqrt{{\hat{h}}_{i}}\right)}^{2}\right]\\ -\sum_{i=0}^{S^{2}}\sum_{j=0}^{B}l_{ij}^{obj}\left[{\hat{C}}_{i}^{j}\log\left(C_{i}^{j}\right)+\left(1-{\hat{C}}_{i}^{j}\right)\log\left(1-C_{i}^{j}\right)\right]\\ -\lambda_{noobj}\sum_{i=0}^{S^{2}}\sum_{j=0}^{B}l_{ij}^{noobj}\left[{\hat{C}}_{i}^{j}\log\left(C_{i}^{j}\right)+\left(1-{\hat{C}}_{i}^{j}\right)\log\left(1-C_{i}^{j}\right)\right]\\ -\sum_{i=0}^{S^{2}}l_{i}^{obj}\sum_{c\in classes}\left[{\hat{p}}_{i}\left(c\right)\log\left(p_{i}\left(c\right)\right)+\left(1-{\hat{p}}_{i}\left(c\right)\right)\log\left(1-p_{i}\left(c\right)\right)\right], \end{array}$$

where $l_{i}^{obj}$ denotes if the target appears in tile *i* and $l_{ij}^{obj}$ denotes that the j−th bounding box predictor in tile *i* is “responsible” for that prediction; $\lambda_{coord},\lambda_{noobj}$ are hyperparameters that separate the loss to loss from bounding box coordinate predictions and that from confidence predictions for boxes that do not contain targets; *S* is the number of grid that input images are divided into; *B* is the number of bounding boxes predicted for each tile (it equals 2 in our study). Note that loss function just penalizes classification error if target appears in that tile. It also penalizes bounding box coordinate error if that predictor is “responsible” for ground truth.

### 3.2. UAV-YOLO: Optimized YOLOv3 for UAV-Viewed Human Detection

**Figure 1:**
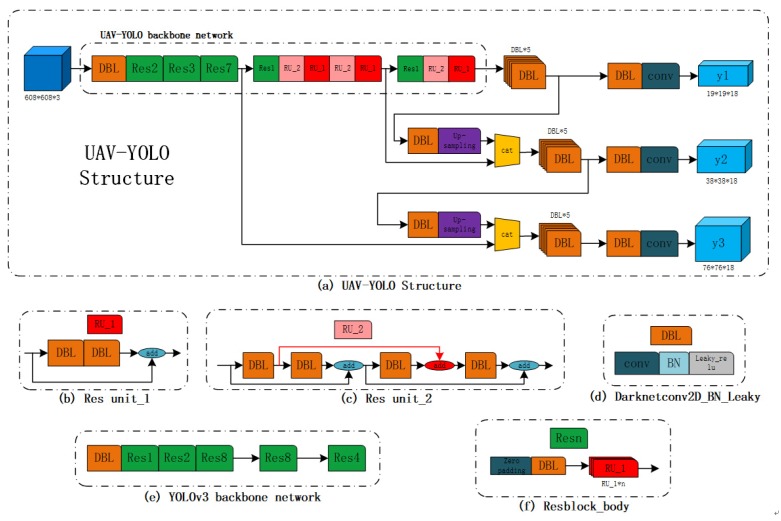
The structure of our proposed UAV-YOLO.

Considering that far distance between target and camera leads to small size of target in UAV perspective scenario, we propose a special detector focusing on UAV perspective based on YOLOv3, named UAV-YOLO, which can enhance the performance on small object detection whilst keeping performance unchanging on normal sized object. The UAV-YOLO structure is shown in Figure 1.

The reason causing small object miss-detection is limited receptive field. To reduce impact of this problem, ResNet unit and backbone network in YOLO are improved. Similar with YOLO, UAV-YOLO uses a single convolutional network which simultaneously predicts multiple bounding boxes and class probabilities for those boxes. It also trains on full images and directly optimizes detection performance. The structure of UAV-YOLO is shown in Figure 1a. Given an image which is resized to 608×608×3 as input to UAV-YOLO, features of input are extracted by the backbone network of UAV-YOLO. To precisely detect different sizes of the targets, 3 different scales of boxes are predicted, which are expressed as y1, y2, and y3 in Figure 1a. In our UAV-viewed dataset experiments, we predicted 3 boxes at each scale, which means the tensor is $N\times N\times [3\dot{(}4+1+1)]$ for the 4 bounding box coordinates, 1 confidence score, and 1 class (human) predictions. Here, *N* is feature maps size of y1, y2, and y3; which are 19, 38, and 76, respectively. From Figure 1a, we can see that y1 is obtained from the end of backbone network which is connected with DBL and a convolutional layer. Here, DBL is combined with a convolutional-layer batch normalization and activated by Leak ReLU, as shown in Figure 1d, and convolutional layer is used to fix y1 size. y2 and y3 are taken from earlier layers of backbone network and merged with our upsampled features using concatenation. It allows us to get more meaningful semantic information from the upsampled feature map. Then, they are also processed by DBL and a few more convolutional layers to predict a similar tensor, although now twice and three times the size compared with y1, respectively.

Compared with YOLOv3, we mainly optimize Res Unit to deep backbone network, which can increase receptive field and provide further benefits for small object detection. Backbone network of original YOLOv3 shown in Figure 1e is mainly made up by Res*n*. Res*n* is the short name of Resblock_body shown in Figure 1f which consists of zero padding, DBL, and Res Unit_1 (named as RU_1 for convenience). *n* is repeat times of RU_1 in the block. RU_1, whose structure is shown in Figure 1b, is the main component of YOLOv3. It is combined with two successive DBLs that use alternate 3×3 and 1×1 convolutional layers and shortcut connection as well to enlarge the receptive field. However, it can get more meaningful semantic information and prevent gradient diffusion by merging more same size convolutional layers. Inspired by this, we design UAV-YOLO using Res Unit_2 (named as RU_2 for convenience) shown in Figure 1c. It is realized by connecting two RU_1s for which convolutional layers have the same output size. Concretely, RU_2 increases another shortcut connection to concatenate two DBLs in different RU_1s. These two connected DBLs are both the first one in those two RU_1s. The structure is shown clearly in Figure 1c, in which the red line with arrow is the added shortcut connect between two RU_1s.

In summary, the main difference between YOLOv3 and UAV-YOLO is the structure of backbone network. Due to our proposed RU_2, backbone network in UAV-YOLO is deeper. In addition, since RU_2 increases extra shortcut connection to concatenate two DBLs in different RU_1s, the repeat of Res Unit in YOLOv3 is also different to that of UAV-YOLO.

**Figure 2:**
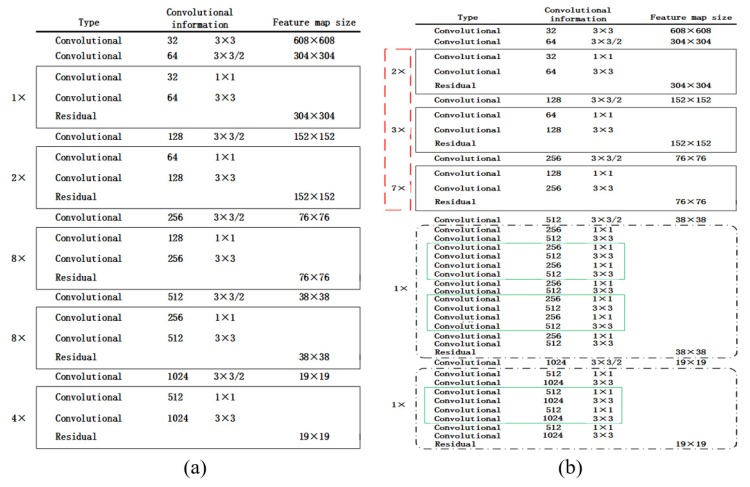
The backbone structure of YOLOv3 and UAV-YOLO. (**a**) Original YOLOv3 structure. (**b**) The proposed UAV-YOLO structure.

The backbone network structure comparison between YOLOv3 (Figure 2a) and UAV-YOLO (Figure 2b) is shown in Figure 2. Black solid rectangle in both Figure 2a,b is RU_1, and blue one in Figure 2b is RU_2. Besides RU_1, UAV-YOLO backbone network also consists of three RU_2 interspersed with RU_1, which is marked by black dotted rectangle in Figure 2b. This kind architecture can enhance the network permutation complexity without extra processing time. In addition, in order to further increase the receptive field of UAV-YOLO, we also change Resblock_body repeat times.

To sum up, UAV-YOLO is optimized based on YOLOv3 at two aspects: (i) shortcut connection is conducted to design Res Unit_2, which is realized by concatenating two DBLs in different Resblocks that have the same output size; (ii) repeat times of each Res Unit is changed to deepen the network structure. It can not only increase receptive filed but also enhance semantic feature extraction ability of the network by fusing these two optimizations.

### 3.3. Optimized UAV-YOLO Training for UAV-Viewed Human Detection

**Figure 3:**
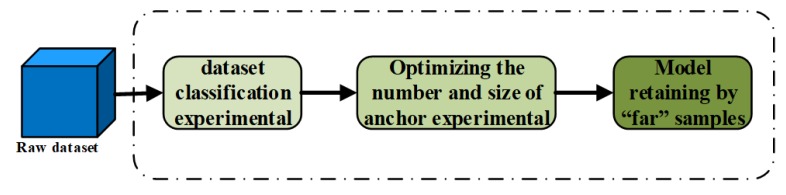
The proposed training method and steps to enhance YOLOv3 performance on UAV-viewed human detection.

In our study, YOLOv3 is first trained by collected UAV-viewed dataset without classifying. Then, we test the detector on test dataset, for which the mean average precision (mAP) is just 51.41% and IOU accuracy is 66.1%. With the aim of enhancing the performance on UAV-viewed human detection, we optimize the YOLO training method as shown in Figure 3.

**UAV-viewed dataset classification.** UAV-viewed dataset includes samples that collect from top view and flat view. In addition, there are also great differences in distance between target and camera. Both these two factors may cause dataset imbalance, which will reflect the detection performance. In our study, we separate collected UAV-viewed dataset into three categories (normal, far, and games) considering distance and background clutters.

**Optimizing the number and size of anchor box by k-means.** K-means algorithm is to separate all samples to *k* clusters, which are usually chosen to be far enough apart form each other spatially, in Euclidean Distance, to produce effective data mining results. The distance calculation function is as follows:

(3) $$d\left(x,y\right)=\sqrt{{\left(x_{1}-y_{1}\right)}^{2}+{\left(x_{2}-y_{2}\right)}^{2}+\cdots +{\left(x_{n}-y_{n}\right)}^{2}},$$

where $x_{n}$ is normalized horizontal axis of bounding box center, $y_{n}$ is normalized vertical axis of bounding box center.

To different dataset organization and detected objects, different anchor mechanisms may influence detector performance a lot. In our study, different number and size of anchor boxes are obtained by k-means. We set 3, 6, and 9 anchor boxes for each target, respectively. Then, k-means is applied to obtain the size of anchor boxes which are imported to network. Based on the experiment, we can find the best number and size of anchor box.

**Model retaining by “far” samples.** Hard sample retaining can effectively enhance the performance of model. In our study, “far” samples are the hard sample due to small size. The network is retained by “far” sample in 5000 iterations.

## 4. Experimental Results

This section shows experimental results of the proposed detection method. We first introduce collected UAV-viewed dataset and implementation detail. Then, we show experimental result on different training method optimization. Finally, experimental result on UAV-YOLO and comparison with other detectors are introduced. We do comparison experiments using a single Nvidia GTX Titan XP GPU with an i7-7820X 3.6 GHz CPU and 64 GB RAM.

### 4.1. UAV-Viewed Dataset and Implementation Detail

Due to the lack of UAV perspective dataset available on the internet, this study collects much more images including UAV123 and shooting by ourselves. It totally includes 4406 images which are separated to training data (3776 images) and test data (630 images) following a ratio of 6:1. They are divided into training/test data following three rules: (1) images in the same video are collected in a discrete way which means that we collect one image with a fixed interval (we collect 1 image in every 5 continuous images); (2) images that are from the same video should be either training data or test data; (3) if there is more than one video from the same flight, they should be separated into training data and test data randomly. The dataset is named as UAV-viewed and labeled by labelImg.

Since we focus on human detection, the UAV-YOLO is first trained by 74,910 human samples, including 8102 samples selected from VOC and COCO. Then, transfer learning is applied based on UAV-viewed dataset. The model is tested by 5000 samples selected from VOC/COCO and our collected UAV-viewed test dataset.

### 4.2. Experimental Results of Optimized Training Method Using YOLOv3

Object detection performance is evaluated by mAP and overlap ratio between ground truth and predicted bounding box (IOU). To speed up model convergence, weight training on ImageNet [38] is used as weight initialization.

**UAV-viewed dataset classification.** Following the UAV-viewed data classification method introduced in Section 3.3, YOLOv3 is trained as described in Section 4.1 and tested on UAV-viewed test dataset. The results are shown in Table 1. It can be viewed that, compared with original UAV-viewed dataset training, classified UAV-viewed dataset training can improve the performance considerably. As shown in Table 1, mAP is increased from 51.41% to 90.88%.

**Figure 4:**
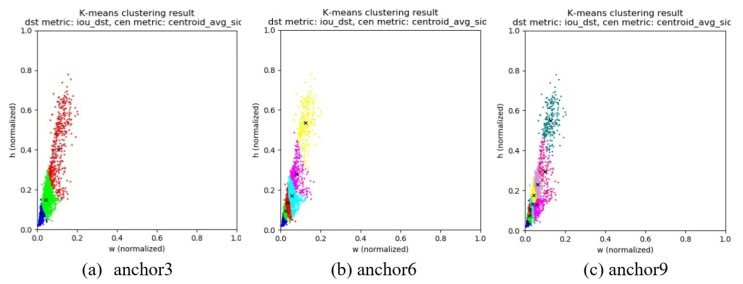
Clustering results on different number of anchor boxes.

**Optimizing the number and size of anchor box by k-means.** As mentioned before, we also optimized the number and size of anchor box by k-means. The anchor box is clustered to 3, 6, and 9 with different size, respectively. This experiment is based on UAV-viewed dataset classification. The results are also shown in Table 1. We find that setting 9 anchor boxes can get the best performance which can reach 80.59% on IOU. In addition, it also works well on the “far” category in which mAP can reach 60.67%. The clustering result on different numbers of anchor boxes is shown in Figure 4, which are named as anchor3, anchor6, and anchor9. The reason that anchor9 works best is that it can increase the receptive field and get more detailed information of the target.

**Model retraining by “far” samples.** The model is retrained by “far” samples, which can enhance the performance on "far" condition with limited decreasing enhancement on “normal” and “games” conditions. It can be viewed in Table 1 that mAP and IOU increase 1.05% and 4.41% for "far" samples; however, mAP just decreases 0.01% and 0.43% for "normal" and "games" samples, respectively.

### 4.3. State-of-the-Art Comparison

After optimized training method confirmation, the proposed detector UAV-YOLO is trained and evaluated. The experiment results are shown in Table 2. It can be viewed that the performance on “far” samples is improved a lot whilst keeping the performance on other conditions almost unchanging. Compared with other detectors including YOLOv3, SSD300, and SSD512, both mAP and IOU of UAV-YOLO increase a lot, reaching 66.25% and 68.86%, respectively.

We also compare our method with other one-stage object detection method on our selected 5000 samples from VOC/COCO. The results are shown in Table 3. It can be seen that our method has better performance and also well-balanced accuracy and processing time.

The purpose of introducing selected human samples form VOC/COCO is to enhance the robustness and generalization of UAV-YOLO. Considering both Table 2 and Table 3, it can be seen that our proposed UAV-YOLO can perform well on both general human dataset and UAV-viewed dataset. It even performs better on both of them compared with YOLOv3.

**Figure 5:**
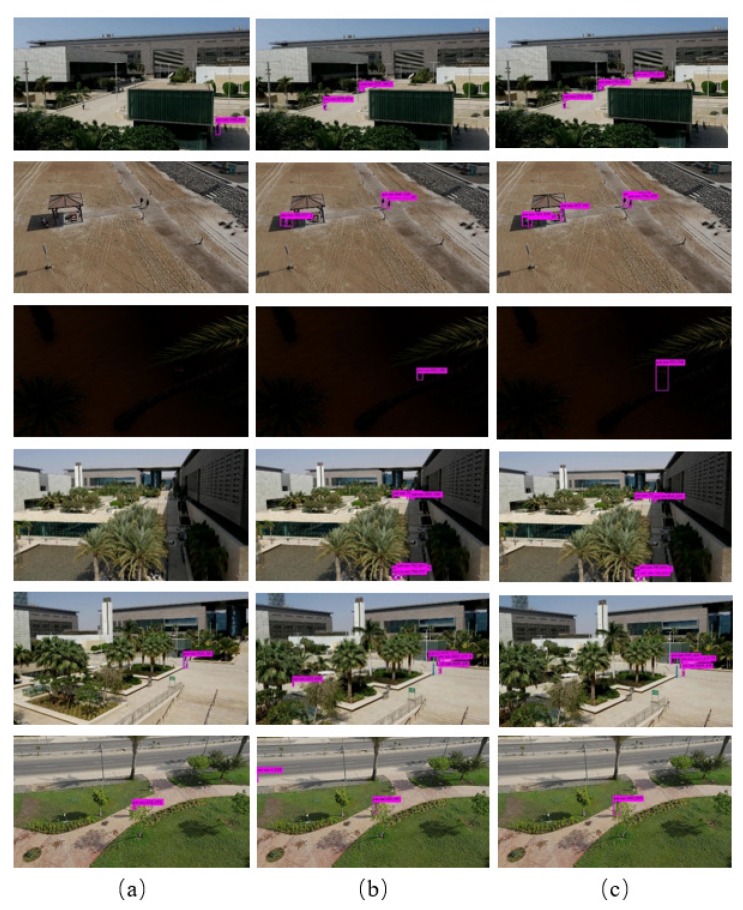
Detected results of different methods on UAV-viewed dataset. (**a**) Detected results of original YOLOv3; (**b**) detected results of YOLOv3 using optimized training method; (**c**) detected results of UAV-YOLO.

An illustration of detected results of original YOLOv3, optimized training method on YOLOv3, and UAV-YOLO for some samples in UAV-viewed is shown in Figure 5. From vision perspective, UAV-YOLO can tackle miss-detection and false detection well. It also can be seen that UAV-YOLO can satisfy object scale variation.

## 5. Conclusions

In this paper, we propose a UAV perspective object detection method based on YOLOv3. We analyze and find the reason causing small object miss-detection and false detection is limited receptive field. To reduce this condition, we first optimize Resblock in darknet by concatenating two ResNet units who have the same width and height. Then, entire darknet structure is improved by increasing convolution operation at early layer to enrich spatial information. It is worth mentioning that both of them can enlarge the receptive field. Besides, we also collect UAV-viewed dataset which is specially used for UAV perspective or small object detection. Based on UAV-viewed dataset, we also develop an optimized training method including training data classification, anchor box confirmation by k-means, and hard data retaining. Representative experimental results compared with other methods demonstrate our method performs well overall in various kinds of challenges, especially in small object detection.

## Figures and Tables

**Table 1:** Mean average precision (mAP) and intersection over union (IOU) performance on UAV-YOLO using different optimized training methods.

	UAV-Viewed		Normal		Games		Far	
Optimized Method	mAP/%	IOU/%	mAP/%	IOU/%	mAP/%	IOU/%	mAP/%	IOU/%
Original data	51.41	66.17	90.81	85.75	94.32	91.44	15.58	29.12
Classified data	90.88	78.20	90.83	80.40	90.76	74.11	56.44	55.02
Anchor3	90.84	79.07	90.86	80.20	90.91	72.62	59.60	55.83
Anchor6	90.88	78.77	90.85	82.13	87.35	71.05	57.15	56.43
Anchor9	90.91	80.59	90.91	84.16	90.91	74.40	60.67	57.43
Mining	90.89	80.29	90.90	83.85	90.48	74.11	61.72	61.84

**Table 2:** mAP and IOU performance comparison on different detectors using our collected UAV-viewed dataset.

	UAV-Viewed		Normal		Games		Far	
Optimized Method	mAP/%	IOU/%	mAP/%	IOU/%	mAP/%	IOU/%	mAP/%	IOU/%
UAV-YOLO	90.86	80.42	90.90	84.11	90.62	76.54	64.42	68.02
YOLOv3	90.89	80.29	90.90	83.85	90.48	74.11	61.72	61.84
SSD300	89.87	72.34	90.68	76.45	89.19	68.21	56.01	52.98
SSD512	90.92	74.23	90.89	78.86	90.71	70.08	61.09	56.84

**Table 3:** Comparison results with state-of-the-art one-stage detection method on selected human samples from VOC/COCO.

Methods	mAP/%	IOU/%	Time/fps
UAV-YOLO	72.54	70.05	20
YOLOv3	72.21	68.43	20
SSD300	62.94	60.72	23
SSD512	72.54	60.72	23
